# Optimizing Mean Arterial Pressure Targets for Septic Shock Patients With Chronic Hypertension: A Narrative Review

**DOI:** 10.1002/hsr2.70696

**Published:** 2025-05-19

**Authors:** Mutaz I. Othman, Emad M. Mustafa, Ahmed E. Abdelwahab, Ali A. Hssain, Abdulqadir J. Nashwan

**Affiliations:** ^1^ Nursing Department Hamad Medical Corporation Doha Qatar; ^2^ Department of Critical Care Hamad Medical Corporation Doha Qatar; ^3^ Department of Public Health, College of Health Sciences QU Health Qatar University Doha Qatar

**Keywords:** acute kidney injury, chronic hypertension, critical care, mean arterial pressure, septic shock

## Abstract

**Background and Aims:**

Septic shock is a serious infection‐related condition that has a big effect on public health. To improve organ perfusion and prognosis in septic shock patients with chronic high blood pressure, optimal mean arterial pressure (MAP) targets are needed. This narrative review aims to summarize existing knowledge and factors to determine the most effective MAP targets in septic shock patients with chronic hypertension.

**Methods:**

A careful review of relevant literature was conducted to understand the factors that affect MAP targets in septic shock patients with chronic hypertension. Long‐term hypertension patients require personalized MAP targets based on age, health conditions, and septic shock severity.

**Results:**

five studies were identified in this narrative review. Guidelines suggest 65‐75 mmHg for most cases, but higher targets may be beneficial for proper organ perfusion. Continuous hemodynamic monitoring allows dynamic adjustment of MAP targets.

**Conclusion:**

It is crucial to implement personalized MAP management strategies to achieve optimal outcomes for patients with chronic hypertension who are experiencing septic shock. However, there is a lack of consensus on optimal MAP targets among patients with chronic hypertension, which may require higher MAP targets to maintain adequate tissue perfusion. An approach that considers each patient's unique characteristics and includes ongoing assessment is critical for achieving the best MAP targets and improving patient prognosis.

## Key Messages

- The review emphasizes the necessity for individualized mean arterial pressure (MAP) targets in septic shock patients with chronic hypertension, considering factors such as age, health conditions, and the severity of septic shock to optimize organ perfusion and patient outcomes.
- While guidelines generally recommend maintaining MAP between 65 and 75 mmHg, there is ongoing debate regarding the potential benefits of higher targets (75 and 85 mmHg) for preventing complications like acute kidney injury, highlighting the need for further research to establish optimal MAP targets.
- The review discusses potential risks associated with aggressive blood pressure management, such as increased incidence of atrial fibrillation, underscoring the importance of balancing treatment benefits and risks in the management of septic shock.

## 1 Introduction

Sepsis is a significant medical condition that arises when the body's immune response to an infection causes extensive inflammation. Septic shock occurs when sepsis progresses to a more severe stage, leading to organ dysfunction or hypoperfusion [1]. Sepsis and septic shock are major global medical concerns. Septic shock has a profound effect on public health, leading to a significant number of illnesses and deaths reported worldwide in annual reports [2]. Despite recent healthcare developments, the mortality rates associated with severe sepsis and septic shock remain unacceptable.

Septic shock may present with symptoms such as hypotension, decreased urine production, respiratory distress, and changes in mental status. Septic shock requires immediate identification, early resuscitation, appropriate antibiotic administration, and comprehensive supportive care [3]. highlighting the urgent need for focused hemodynamic interventions [4]. Exploring the area of hemodynamic support via the administration of fluids and vasopressors. Septic shock is characterized by three major pathophysiological consequences for the cardiovascular system, including vasodilation, inequitable blood flow distribution, and reduced heart function [5]. Fluid resuscitation, inotropic treatment, and vasopressor therapy are the main techniques used to support hemodynamics in septic shock patients [6].

Globally, arterial blood pressure has been a traditionally established target. When determining the optimal mean arterial pressure (MAP) targets in septic shock, several factors need to be taken into consideration [7]. These include the patient's baseline blood pressure, the presence of comorbidities such as hypertension or cardiovascular disease, the severity of the illness, the response to fluid resuscitation, and the adequacy of tissue perfusion. Nowadays, studies indicate that instead of relying on fixed targets, it could be more beneficial to create personalized blood pressure goals based on dynamic factors such as MAP and arterial waveform analysis.

Chronic hypertension can have a significant impact on vascular physiology and autoregulatory mechanisms, making blood pressure management more challenging in cases of septic shock [8]. The guidelines for shock advise maintaining a MAP of at least 65 mmHg when using vasopressor therapy. Additionally, it was previously proposed that patients with already‐present chronic hypertension and atherosclerosis may benefit from higher MAP targets. The review addresses the effectiveness and efficacy of various MAP targets. Having an effective understanding of MAP management in this complex set of patients is essential for clinicians to adeptly customize treatment strategies [9]. Recognizing that one approach may not be ideal for every patient, personalized methods of managing arterial blood pressure targets in septic shock have gained significant popularity. The severity of septic shock may fluctuate, leading to hemodynamic instability, organ failure, and different levels of therapeutic response. Thus, customizing blood pressure management according to individual characteristics and treatment responses may lead to enhanced patient prognosis. This review aims to summarize existing knowledge and factors to determine the most effective MAP targets in septic shock patients with chronic hypertension.

## 2 Methods

### 2.1 Search Strategy

The literature search involved exploring published articles relating to the area of target MAP septic shock patients with chronic hypertension. The searches involved the utilization of the database which included Embase, Medline, and PubMed. Besides that, the Google Scholar search engine was used to select further studies. Ethical approval details and informed consent are not applicable for this study.

This narrative review did not utilize formal validated models such as PRISMA or AMSTAR; however, it adhered to a rigorous and transparent methodology for identifying and synthesizing relevant studies. Two independent reviewers conducted a literature screening to ensure consistency and reduce bias according to the subsequent inclusion criteria: studies published between 2014 and 2024, those research “septic shock.” “Mean arterial pressure targets” and “chronic hypertension,” Next, compile synonyms and related terms, such as “personalized approach,” “blood pressure management,” and “sepsis‐induced hypotension.” Combine these keywords using Boolean operators (AND, OR) to construct search queries.

The abstracts of all those studies which that were identified by the search strategies were examined, works in English, and a full‐text version of those which achieved the eligibility criteria was then obtained. Moreover, reference lists from chosen papers were checked for additional related studies. As shown in Table 1 several studies were identified, but few met the criteria, and two reviewers, the second reviewer aimed to ensure transparency in the study selection process and maintain the reliability of the review results.

**Table 1:** Search strategy and search operators used.

Query	Result
(Chronic hypertension OR (mean arterial pressure targets OR blood pressure management)) AND (septic shock OR sepsis‐induced hypotension)	437
((Chronic hypertension AND blood pressure management) OR (septic shock)) AND (mean arterial pressure targets)	196
(Chronic hypertension) AND (septic shock) AND (mean arterial pressure targets)	14
((Chronic hypertension AND blood pressure management) AND (septic shock))	5

## 3 Result

Five studies identified in this narrative review areshown in Table 2. The study discusses the importance of MAP targets in patients with septic shock, particularly those with chronic hypertension. The search result involved conducting a review of existing literature that included two comparative studies, and three observational studies.

**Table 2:** Summary of studies included in the literature review (*n* = 5).

*Ref*.	*Study objectives*	*Sample characteristics*	*Methodology*	*Main finding*	*Limitations*
Corrêa, T.D., S.M. Jakob, and J. Takala [10]c,2015	The study objectives are to discuss the need for individualized resuscitation targets based on the patient's baseline condition and to present directions for future research on the impact of individualized MAP targets on various physiological parameters and outcomes in septic shock patients.	Patients with chronic hypertension ‐ More than 40% of patients in the septic shock trial had a history of chronic hypertension	The study assessed the effects of norepinephrine administration on systemic hemodynamic, tissue perfusion, and sublingual microcirculation in septic shock patients with chronic hypertension, a single‐center prospective open‐label study without a concurrent control group.	Norepinephrine administration improved sublingual microcirculation but did not improve systemic tissue perfusion. Higher mean arterial blood pressure (MAP) levels in septic shock patients did not improve survival or renal replacement therapy but decreased it in chronic hypertension patients.	Late resuscitation phase, short interval between measurements, and lack of a concurrent control group may affect results and limit definitive conclusions.
Asfar, P., et al. [11], 2014.	The study objective was to compare the effectiveness of targeting a mean arterial pressure of 80 to 85 mmHg with targeting 65 to 70 mmHg in patients with septic shock undergoing resuscitation.	Patients with septic shock and patients with chronic hypertension	The methodology involved a multicenter, open‐label trial where 776 patients with septic shock were randomly assigned to different mean arterial pressure target groups, with the primary endpoint being mortality at day 28.	There was no significant difference in mortality at either 28 or 90 days between patients with septic shock undergoing resuscitation with a mean arterial pressure target of 80 to 85 mmHg compared to 65 to 70 mmHg.	The study found no significant difference in mortality between high and low blood‐pressure target groups, higher incidence of atrial fibrillation in high‐target groups, and no association between renal‐replacement therapy and mortality.
Beloncle, F., et al. [11], 2016	The study objectives are to review the physiological rationale and clinical evidence for increasing mean arterial pressure in septic shock.	Patients with septic shock and patients with chronic hypertension	The SEPSISPAM Trial, a large prospective study, found that high mean arterial pressure may prevent acute kidney injury, supported by observational and interventional studies.	Studies suggest higher mean arterial pressure may prevent acute kidney injury in some patients. The SEPSISPAM Trial found no difference in mortality between high and low arterial pressure targets in septic shock patients.	The study lacks definitive answers, shows no mortality difference between high and low mean arterial pressure groups, and significant renal failure difference only in chronic hypertension patients.
*paper*	***Study objectives***	***Sample characteristics***	***Methodology***	***Main finding***	***Limitations***
Kuragayala, S.D., et al. [12], 2019	The study objective is to evaluate the effects of two different mean arterial blood pressure (MAP) targets in septic shock.	Patients admitted to the ICU with sepsis, older patients with septic shock	The methodology involved a prospective observational study enrolling 100 patients with septic shock in the ICU, divided into two groups to assess the effects of different mean arterial blood pressure targets.	Maintaining a MAP above 65 mmHg is crucial to reduce mortality in septic shock, with a target range of 75–85 mmHg potentially reducing the incidence of acute kidney injury in patients with chronic hypertension. Prolonged time below 65 mmHg increases the risk of mortality and organ injury.	The study, which was conducted using an observational design and a single‐center setting, lacked exploration of long‐term outcomes and did not consider comorbidities.
Xu, J.‐Y., et al. [13], 2015	To assess the effect of mean arterial pressure titration to a higher level on microcirculation in hypertensive septic shock patients.	The study analyzed patients with septic shock for less than 24 h, a history of hypertension, and found a 53% in‐hospital mortality rate due to pneumonia.	A single‐center study enrolled hypertensive septic shock patients after fluid resuscitation and norepinephrine use, titrated mean arterial pressure, and evaluated sublingual microcirculation using side stream dark field imaging.	The study found that elevating mean arterial pressure from 65 mmHg to normal levels is linked to enhanced microcirculation in patients with hypertensive septic shock.	The study's limitations include the use of the NICOM system for cardiac output monitoring, absence of a control group, and lack of normotensive controls.

The study investigates the impact of norepinephrine administration on the systemic hemodynamic, tissue perfusion, and sublingual microcirculation of septic shock patients with chronic hypertension found that increasing MAP improved sublingual microcirculation but did not significantly improve systemic tissue perfusion [10]. However, the high‐target group exhibited a higher incidence of atrial fibrillation, along with indicating potential risks associated with aggressive blood pressure management.

Another study also reviewed physiological rationale and clinical evidence for increasing MAP in septic shock, emphasizing the lack of consensus on optimal targets. The SEPSISPAM trial found no mortality difference between high and low MAP groups [14]. The review advocates for a MAP target of 65–75 mmHg in septic shock patients but suggests a higher MAP may be beneficial in preventing acute kidney injury in patients with chronic hypertension.

An observational study suggested that maintaining a MAP above 65 mmHg is crucial for reducing mortality in septic shock, while a target range of 75–85 mmHg may further reduce the incidence of acute kidney injury in patients with chronic hypertension [12]. A high mean arterial pressure target is associated with improved microcirculation in hypertensive septic shock patients with previous hypertension, but limitations include the absence of a control group and potential confounding factors. The study evaluates the impact of two mean arterial blood pressure targets on septic shock mortality in India. Maintaining a MAP above 65 mmHg reduces mortality and acute kidney injury in patients with chronic hypertension.

A multicenter, open‐label trial aimed to compare the effectiveness of targeting 80 to 85 mmHg with 65 to 70 mmHg in patients with septic shock undergoing resuscitation [15].

The study found no significant difference in mortality rates between patients with septic shock undergoing resuscitation and those with a mean arterial pressure target of 65 to 70 mmHg. The primary endpoint was mortality at day 28.

The study involved a single‐center, open‐label approach, focusing on patients with a history of previous hypertension, a mean duration of 16 h, and a 53% in‐hospital mortality rate. A study involving hypertensive septic shock patients found that increasing mean arterial pressure from 65 mmHg to normal levels improved microcirculation [13]. The most common source of sepsis was pneumonia. The study highlights the importance of titration in assessing microcirculation in hypertensive septic shock patients.

## 4 Discussion

The discussion section of this study focuses on the complex relationship between MAP targets and outcomes in patients with septic shock, particularly those with underlying chronic hypertension. It highlights the conflicting findings from different studies regarding optimal MAP targets and the lack of consensus in the field. The discussion also acknowledges the potential risks associated with aggressive blood pressure management and emphasizes the need for individualized approaches based on patient characteristics and response to treatment indicating several key themes emerge. Table 3 distinguishes between the advantages of keeping MAP above and below 75 mmHg. After analyzing the available studies, it was determined that MAP of 75 mmHg is possible to consider as an average value for those populations.

**Table 3:** Advantages and disadvantages of MAP targets in septic shock management patient with chronic hypertension.

Factors	Advantages	Disadvantages
**MAP 75–85 mmHg**	o Higher MAP targets may reduce mortality and prevent complications like acute kidney injury. o May be sufficient to meet tissue perfusion and microcirculation.	o Significantly a higher incidence of atrial fibrillation.
**MAP 65–75 mmHg**	o Lower incidence of adverse events associated with aggressive blood pressure management.	o May not adequately improve tissue perfusion in all patients. o Potential for inadequate microcirculation improvement.

### 4.1 The Importance of Individualized Care

The studies emphasize the importance of customized strategies for MAP in patients with septic shock. Several factors play a crucial role in determining the ideal targets for MAP, such as chronic hypertension and the response of patients to treatment. Although there is no Consensus agreement, the studies provide support for implementing personalized approaches to managing MAP. Personalized MAP strategies are a paradigm shift in treating septic shock, especially for patients with chronic hypertension [16]. By tailoring hemodynamic support to individual patients' unique physiological needs, these strategies can improve outcomes and reduce complications. Personalized MAP targets can optimize organ perfusion, reduce complications, and enhance precision with emerging technologies [17]. Noninvasive monitoring technologies like sublingual video microscopy and renal Doppler ultrasound can provide real‐time feedback on microcirculatory and organ‐specific perfusion. Risk stratification tools can help identify patients at higher risk for complications and guide MAP titration [18].

**Figure 1:**
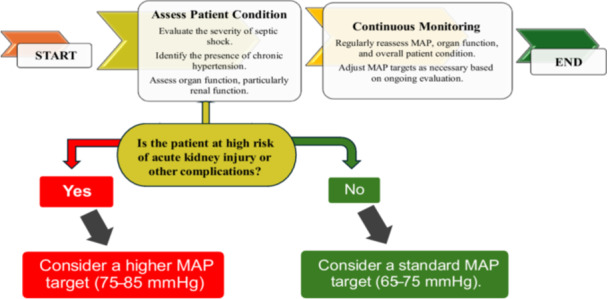
Decision‐making Process MAP Targets in Septic Shock Management for Patients with Chronic Hypertension.

As presented in Figure 1, considering patient characteristics and treatment responses is crucial when making decisions. It seems that there is a growing trend towards personalized medicine in the management of septic shock [19].

Emerging technologies, like noninvasive cardiac output monitors and AI‐driven predictive models, help doctors decide what MAP targets to use in people who are in septic shock and have high blood pressure all the time [20]. Such tools deliver real‐time hemodynamic information, enhance organ perfusion, and reduce complications. Biomarkers indicative of endothelial dysfunction and microcirculatory impairment facilitate accurate adjustments in MAP targets [21]. This data‐driven approach changes the focus from a standard approach to a more stratified, patient‐centred model. This makes hemodynamic support better and opens the door to more research in the future.

A practical decision‐making algorithm for managing MAP targets addresses controversies by integrating baseline assessment, dynamic monitoring, risk stratification, and iterative titration need to be considered. This algorithm presented in Figure 1 may guide clinicians in implementing personalized MAP management at the bedside, bridging the gap between research and practice.

### 4.2 Comparing Microcirculation and Microcirculation Perfusion

There is a noticeable distinction between the enhancement of sublingual microcirculation as blood pressure rises and the limited improvement seen in overall tissue perfusion. There is a complex relationship between blood pressure management and tissue perfusion at different levels [22]. Tissue hypoxia or dysoxia, characterized by reduced vascular tone, myocardial depression, and changes in blood flow distribution, is the result of septic shock [23]. Microvascular changes also happen, characterized by reduced blood flow, and heightened vascular permeability. Clinical interventions aim to restore micro‐damages of immune system dysregulation by treating macro‐haematological variables. Treating acute circulatory failure resulting from septic shock promptly is essential to avoid multiorgan dysfunction, a significant therapeutic challenge. Monitoring acute circulatory dysfunction should prioritize the correlation between macro‐ and micro‐responses to shock.

### 4.3 Debate Persists Regarding the Best MAP Targets

Despite thorough investigation, there remains a lack of consensus regarding the optimal MAP targets for individuals suffering from septic shock. There is a continuous discussion surrounding the ideal MAP target for individuals with chronic hypertension. Particularly there is ongoing debate among experts regarding the optimal MAP range to prevent acute kidney injury. Some argue for a range of 65–75 mmHg, while others suggest higher targets [12, 14]. On another hand, some studies suggest that maintaining a higher MAP, possibly in the range of 75–85 mmHg, could be beneficial for preventing complications such as acute kidney injury in this population The study highlights the ongoing debate surrounding MAP targets in septic shock and suggests personalized approaches based on patient response [10, 15]. A meta‐analysis of three randomized controlled trials suggests that further trials are necessary to determine if higher and lower MAP targets in vasodilatory shock significantly differ in mortality [7].

### 4.4 Potential Risks of Aggressive Blood Pressure Management

Potential risks can arise from aggressive blood pressure management, such as an increased likelihood of developing atrial fibrillation. It is important to thoroughly evaluate the benefits and limitations of treatment strategies [14]. Some argue that establishing higher blood pressure targets could potentially increase mortality in patients receiving vasopressor treatment for more than 6 h. On the other hand, lower blood pressure targets did not show any association with adverse events in any subgroup, including patients with chronic hypertension [24].

The argument highlights the current discussion surrounding MAP targets in septic shock. Various studies have provided different conclusions, highlighting the need for further research, such as randomized controlled trials, to determine the optimal MAP targets and their impact on patient prognosis.

The review identifies significant gaps in the literature regarding MAP targets for chronic hypertension, indicating a lack of consensus and insufficient evidence about the potential benefits of higher targets for patients. Current study evidence frequently emphasizes macrocirculatory parameters while overlooking microcirculatory perfusion. Additionally, a lack of comprehensive data regarding long‐term outcomes and the variability within patient populations complicates the assessment of the effects of MAP targets on organ function and quality of life.

The result of this review highlights the complexity of managing blood pressure in patients with septic shock and chronic hypertension. It also emphasizes the significance of stratified treatment strategies according to clinical evidence and the unique characteristics of each patient.

## 5 Conclusion

The current knowledge suggests a MAP target range of 65–75 mmHg for patients with septic shock, including those with chronic hypertension. However, there is a lack of consensus on optimal MAP targets among patients with chronic hypertension, which may require higher MAP targets to maintain adequate tissue perfusion. An individualized approach is critical, considering factors such as septic shock severity, comorbidities, organ dysfunction, and the initial resuscitation response. Balancing benefits and risks are essential for optimizing patient outcomes. Furthermore, randomized controlled trials to compare mean arterial pressure (MAP) targets in people with long‐term high blood pressure, microcirculatory studies to investigate the association between MAP targets and perfusion, and predictive modeling using machine learning methods are all areas that need to be considered in future research. Along with the need to provide definitive evidence and guidance for clinical practice.

The latest research indicates that patients should have individualized targets that take into consideration their unique characteristics [25]. While evidence suggests the potential benefits Despite extensive research and efforts to identify individualized MAP targets in septic shock management, finding the optimal approach continues to be complicated. Further investigation is needed to better understand how patient‐specific factors influence MAP targets and their implications for clinical prognosis. A personalized approach to hemodynamic management holds promises in improving patient care and reducing mortality in septic shock.

## Author Contributions

conceptualization: M.I.O., A.E.A. data curation: M.I.O., A.E.A. Formal analysis: M.I.O., E.M.M., A.E.A. funding acquisition: M.I.O. methodology: M.I.O., A.J.N. Project administration: M.I.O. visualization: M.I.O. writing – original draft: M.I.O., E.M.M. writing – review and editing: A.A.H., A.J.N. All authors have accepted responsibility for the entire content of this manuscript and approved its submission.

## Conflicts of Interest

The authors have no conflicts of interest to disclose. Abdulqadir Nashwan is an Editorial Board member of Health Science Reports and a coauthor of this article. To minimize bias, they were excluded from all editorial decision‐making related to the acceptance of this article for publication.

## Data Availability

Data sharing not applicable to this article as no datasets were generated or analysed during the current study. All authors have read and approved the final version of the manuscript. The lead author [Mr. Mutaz Othman] had full access to all of the data in this study and takes complete responsibility for the integrity of the data and the accuracy of the data analysis.
